# Supplementary material for: Intrabody-Induced Cell Death by Targeting the T. brucei Cytoskeletal Protein TbBILBO1
Source: Microbiol Spectr. 2021 Oct 27;9(2):e00915-21. doi: 10.1128/Spectrum.00915-21 (PMC8549753; doi:10.1128/Spectrum.00915-21)
Supplement: SUPPLEMENTAL FILE 1 — Supplemental material. Download SPECTRUM00915-21_Supp_1_seq13.pdf, PDF file, 7.3 MB [file spectrum00915-21_supp_1_seq13.pdf]

Supplementary Figure. 1

A

Production and purification of **Nb48**: Coomassie gel

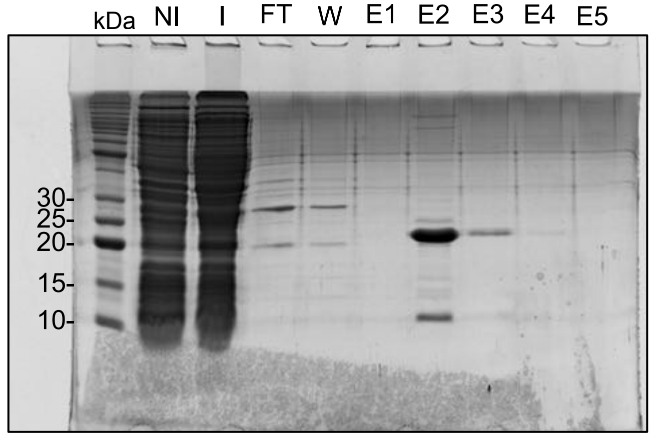

B

Production and purification of **Nb9**: Coomassie gel

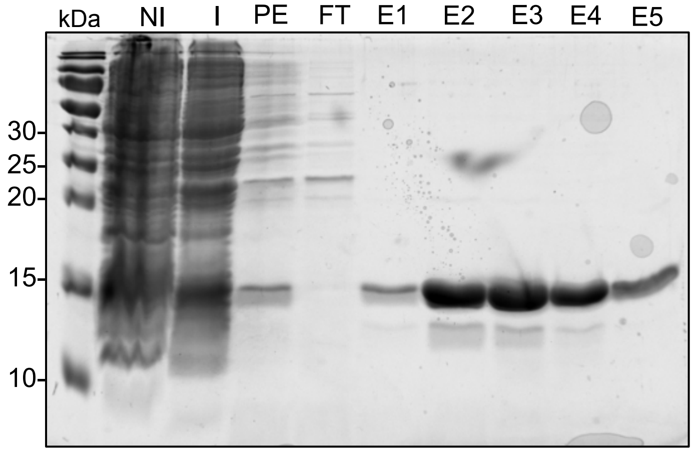

C

Production and purification of **Nb73**: Coomassie gel

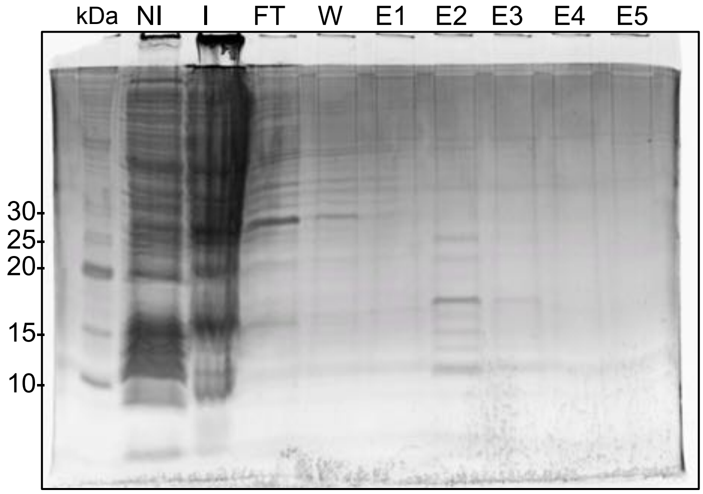

Sup Fig 1. Production and purification of Nb48, 9 and 73

(A) Coomassie blue stained gel of Nb48 expression before (NI) and after IPTG induction (I) and purification samples. Nb48 was obtained from elutions E2-E5. (FT = flow through, W = wash and E1-E5 = elution 1 to elution 5) in bacteria. **(B)** Coomassie blue stained gel of Nb9 expression in bacteria before (NI) and after IPTG induction (I) and purification. Nb9 was obtained from elutions E1-E5 and peaking at E3. (PE = periplasmic extract, FT = flow through and E1-E5 = elution 1 to elution 5). (C) Coomassie blue stained gel of Nb73 expression in bacteria before (NI) and after IPTG induction (I) and purification. Nb73 was obtained from elutions E2-E3. (FT = flow through, W = wash and E1-E5 = elution 1 to elution 5).

Supplementary Figure. 2

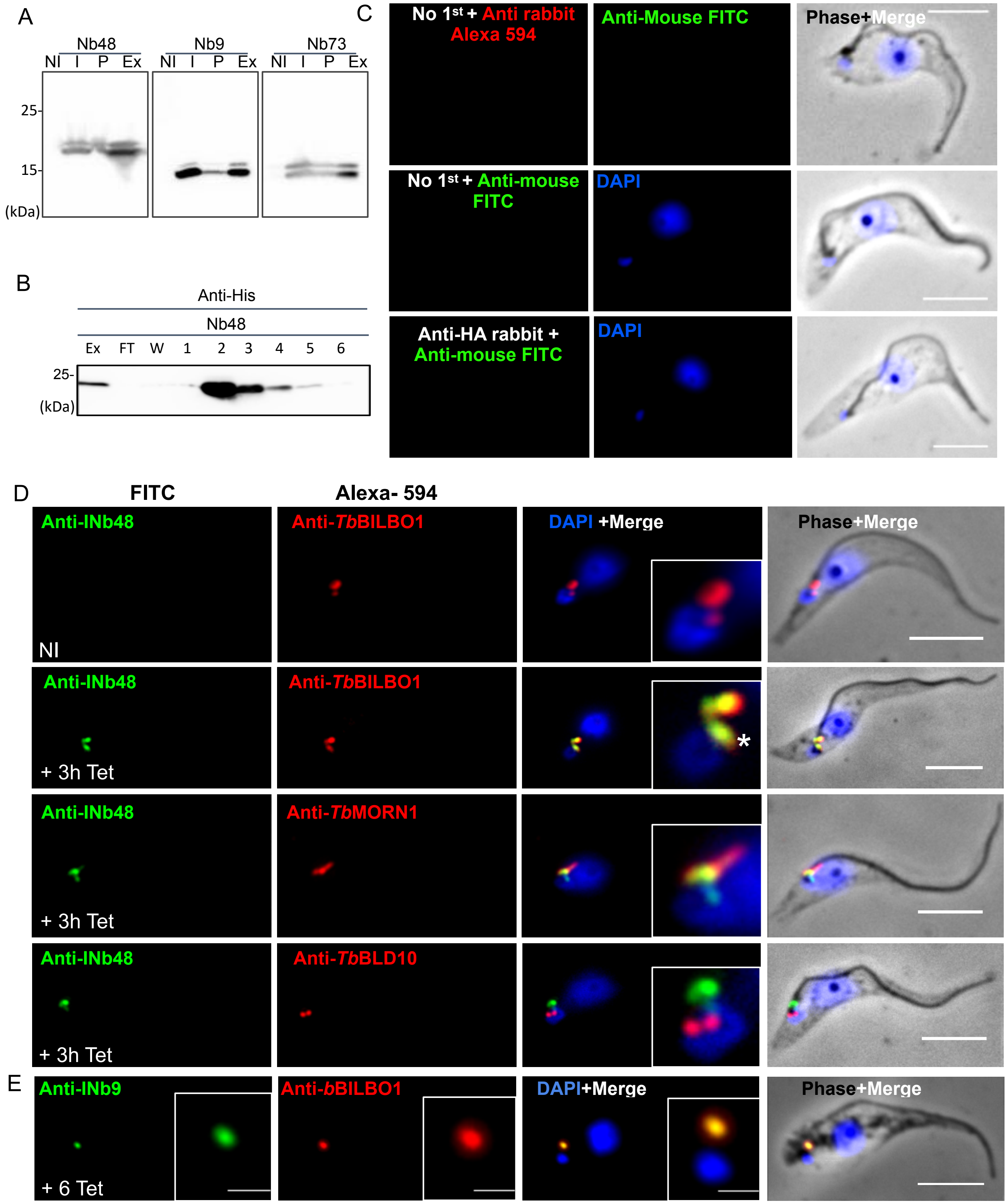

Sup Fig. 2. Anti-*Tb*BILBO1 nanobodies: Western blotting, purification and use in IFA

(A) Bacterial expressed nanobody ($Nb_{::HA::6His}$) samples probed on a WB with anti-HA. Samples of non-induced (NI), induced (I) bacterial cultures, pellet (P) and periplasmic extract (Ex). (B) Purification of $Nb48_{::HA::6His}$: flow through (FT), wash (W) and elutions (lanes 1-6), all lanes were probed by WB using anti-His. (C) IFA controls for (Figure 2A-D and Figure 1). First panel is a WT *T. brucei,* procyclic, cytoskeleton probed with anti-rabbit Alexa fluor 594 followed by anti-mouse FITC but no primary antibodies, demonstrating that there is no cross-reaction between these secondary antibodies or on the cytoskeleton. The second panel is a negative control of WT cytoskeleton probed with anti-mouse FITC but no primary antibody, which demonstrates that there is no cross-reaction between this secondary antibody and the cytoskeleton. The third panel is a negative control of a WT cytoskeleton probed with anti-HA rabbit followed by anti-mouse FITC, which demonstrates that there is no cross-reaction between these antibodies on the cytoskeleton. (D) In non-induced $INb48_{::3cMyc}$ cells (first panel), anti-cMyc reveals no labeling. After $INb48_{::3cMyc}$ expression is induced for 3 hours (+3h; second panel) labelling is visible at the FPC (green) co-localizing with *Tb*BILBO1 (red) at the FPC and along the MtQ (*). The two other panels show localization of $INb48_{::3cMyc}$ (green) with *Tb*MORN1 (red) and *Tb*BLD10 (a basal body marker; red) respectively. (E) After 6 hours of INb9::3cMyc induction INb9 labelling is visible at the FPC (green) and co-localizes with *Tb*BILBO1 (red) at the FPC. Scale bar = 5µm, inset = 1µm.

Supplementary Figure. 3

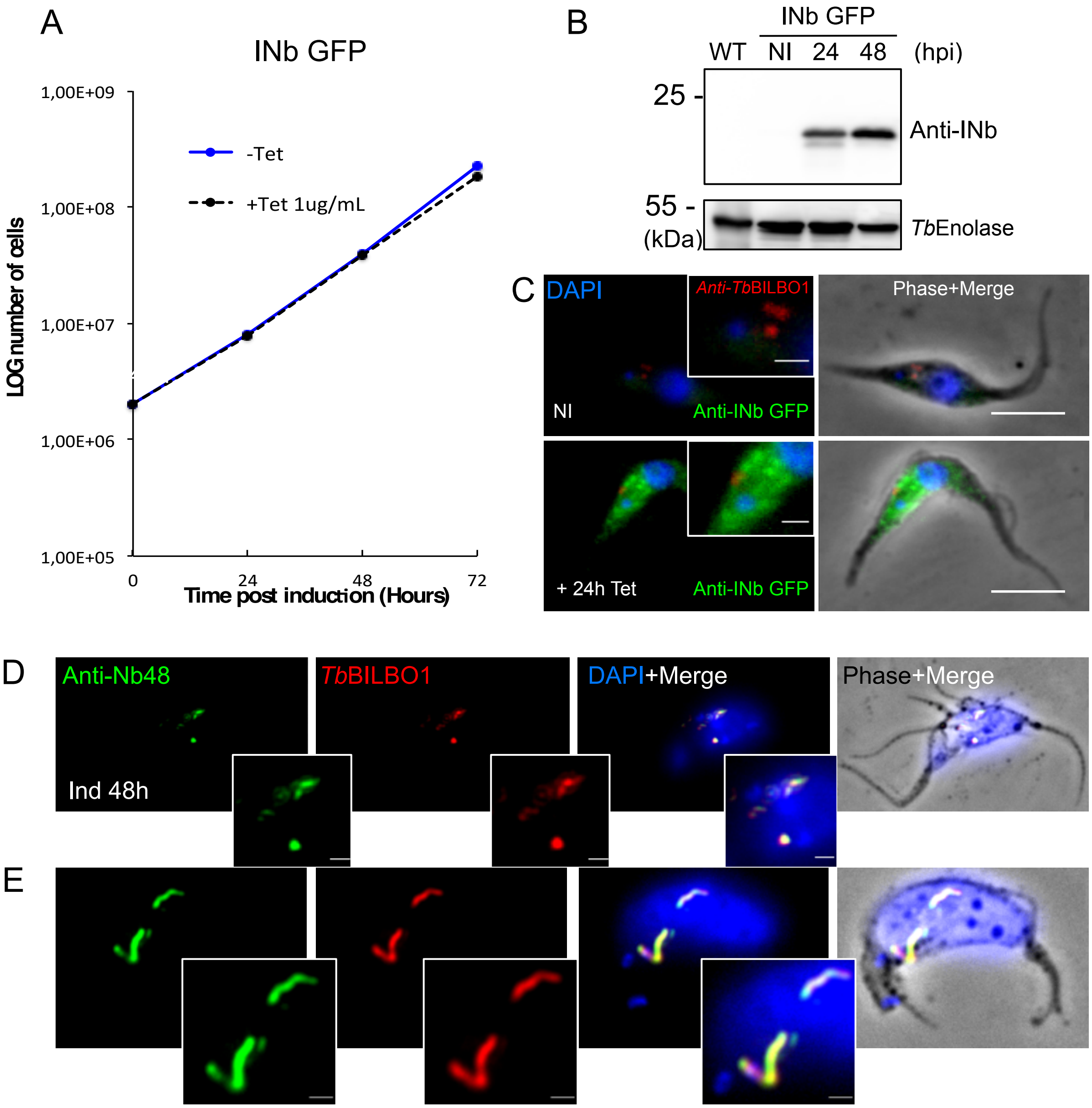

Sup Fig 3. Expression of control anti-GFP nanobody and 48h expression of INb48

(A) Growth curve of *T. brucei* PCF cells expressing INbGFP$_{::3cMyc}$, show that expression of anti-GFP intrabody does not induce a cell growth phenotype. (B) Western blot of expression of INbGFP$_{::3cMyc}$ in *T. brucei* showing expression at 24 and 48hpi. *Tb*Enolase, is a cytoplasmic protein, that has been used as a loading control. (C) IFA of non-induced PCF *T. brucei* cells probed with anti-*Tb*BILBO1 (red) and anti-cMyc to label INbGFP$_{::3cMyc}$ (green - weak background signal) (non-induced NI; top panel). A trypanosome induced for 24h (+Tet) demonstrating cytoplasmic localisation of INbGFP$_{::3cMyc}$ (green) and the normal FPC localisation of *Tb*BILBO1 (red) (Bottom panel). Scale bar = 5µm, in the insets the scale bar = 1µm. (D) IFA of a *T. bruce*i PCF cell expressing INb48$_{::3cMyc}$ for 48h and probed with anti-cMyc (green) and anti-BILBO1 (red). Multiple detached flagella are observed in a single cell, with *Tb*BILBO1 labelling at two disrupted FPs. (E) As in D, but showing two very distorted FPC that consist of long polymers rather than the distinctive annular structure. For the sake of convenience, the protein being probed is named as anti-Nb, plus the relevant nanobody name rather than its tag. Scale bar = 5µm, inset = 1µm.

Supplementary Figure. 4

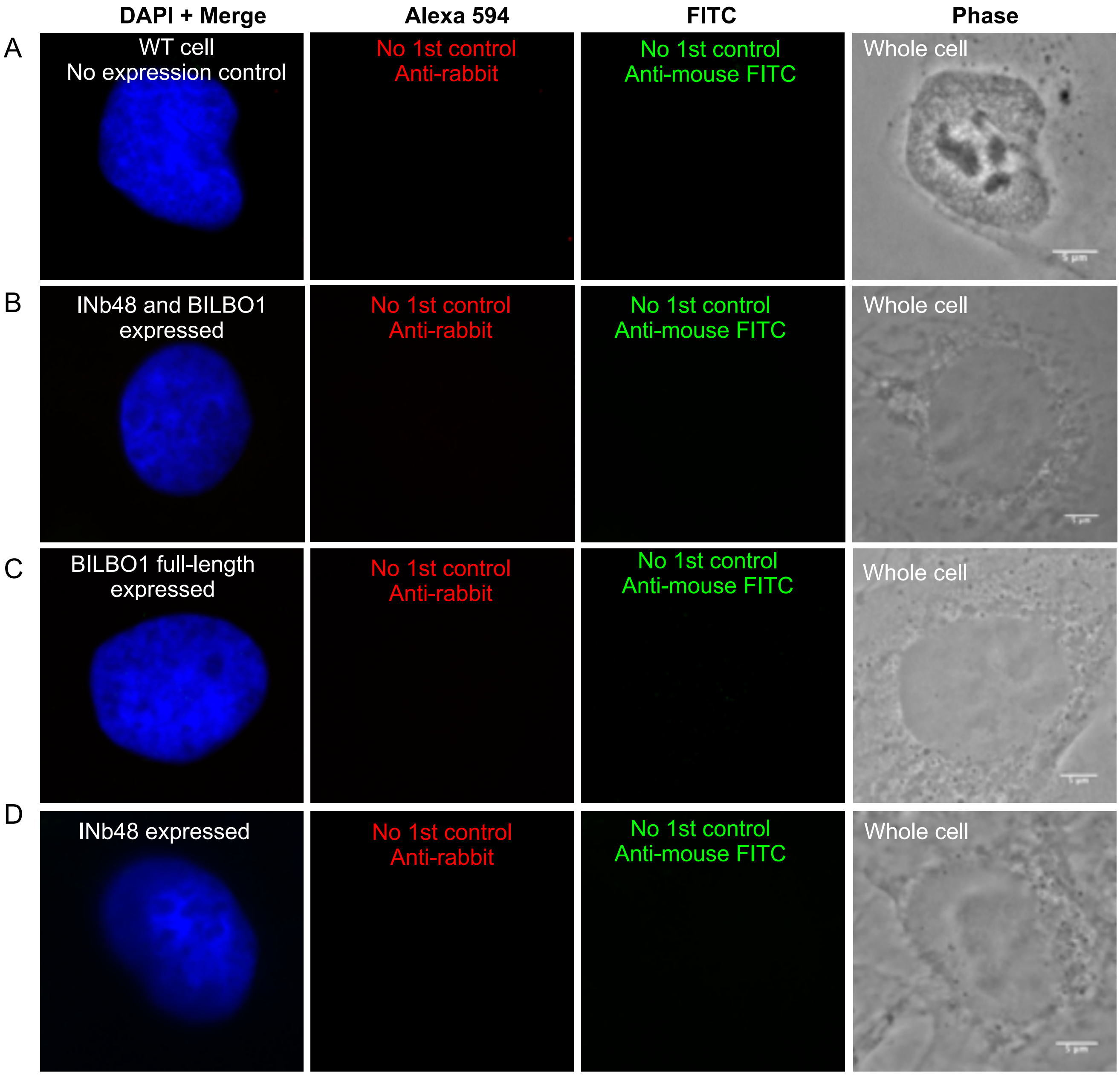

Sup Fig 4. IFA antibody controls for expression of INb48$_{::3HA}$ and/or *Tb*BILBO1 in U-2 OS cells

Controls for Figure 8. (A) Images of a U-2 OS wild-type (WT) whole cell that has not been transfected with INb48$_{::3HA}$ or *Tb*BILBO1 and is probed with secondary antibodies only. No signal is observed. (B) Images of a U-2 OS whole cell that has been transfected with INb48$_{::3HA}$ and *Tb*BILBO1 and probed as in (A); no signal is observed. (C) Images of a U-2 OS whole cell that has been transfected with *Tb*BILBO1 full-length and probed as in (A); no signal is observed. (D) Images of a U-2 OS whole cell that has been transfected with INb48$_{::3HA}$ and probed as in (A); no signal is observed. Scale bars = 5µm.

Supplementary Figure. 5

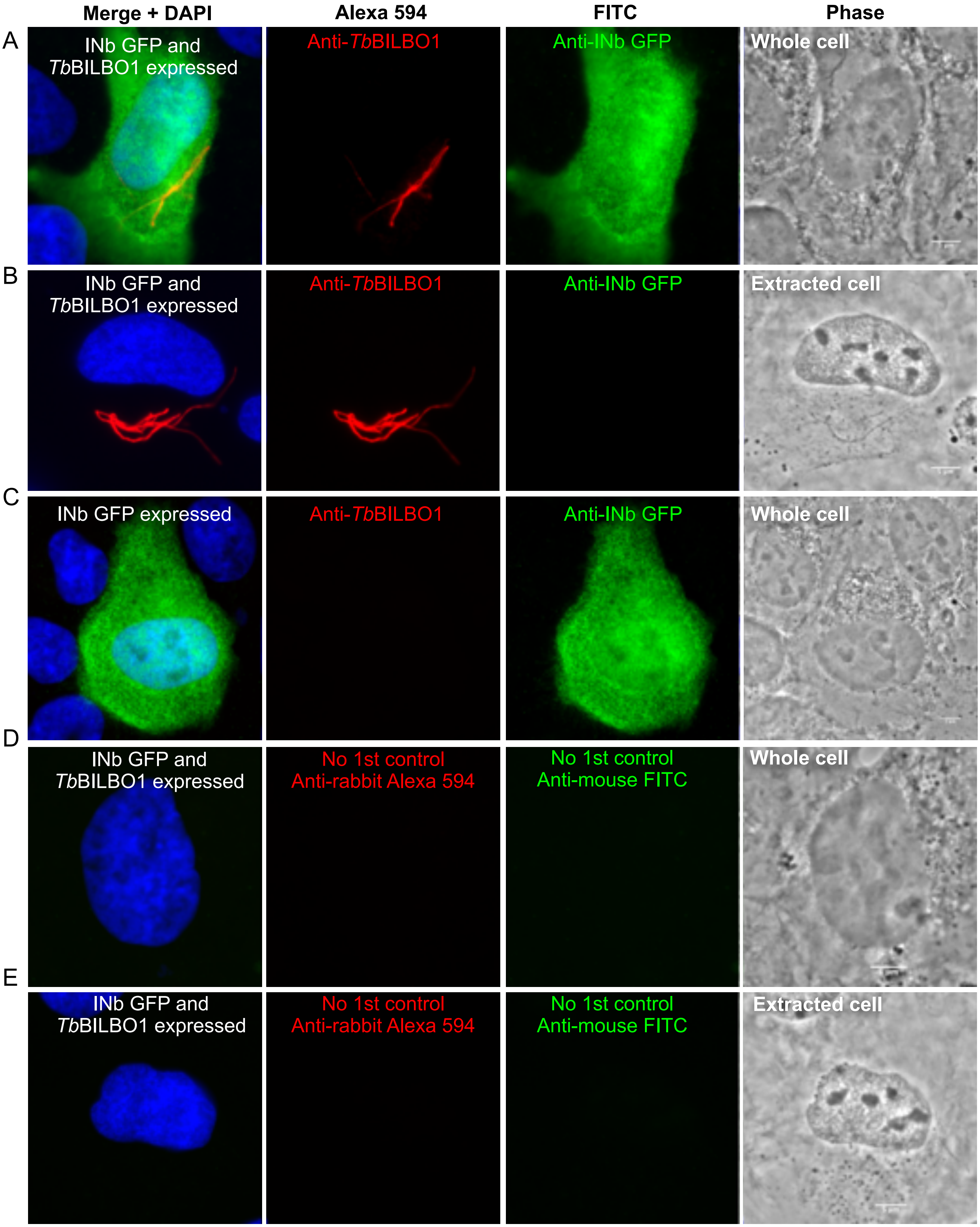

Sup Fig 5. Controls of intra-nanobody anti-GFP ($INbGFP_{::3HA}$) and/or *Tb*BILBO1 expressed in U-2 OS cells

(A) Images of antibody controls of intrabody anti-GFP ($INbGFP_{::3HA}$) and *Tb*BILBO1 expressed in U-2 OS cells and probed with anti-HA, plus anti-*Tb*BILBO1 (1-110) followed by their respective secondary antibodies anti-rabbit Alexa 594 and anti-mouse FITC. Both *Tb*BILBO1 polymers and the cytoplasmic nanobody ($INbGFP_{::3HA}$; green) are observed, but the GFP intrabody does not bind to *Tb*BILBO1 positive polymers (red). (B) Images of a cell treated and probed as in (A) but detergent extracted, illustrating that the anti-GFP intrabody is cytoplasmic and soluble and is removed by detergent extraction, but not the *Tb*BILBO1 polymers (red). (C) Images of a U-2 OS cell only expressing the $INbGFP_{::3HA}$ but probed as in (A) and illustrating a green cytoplasmic labelling of the intrabody only, which does not form polymers. (D and E) Images of antibody controls of $INbGFP_{::3HA}$ and *Tb*BILBO1 expressed in U-2 OS cells and probed with secondary antibodies only: Alexa 594 and anti-mouse FITC. (D) is a whole cell whilst (E) is detergent extracted. No signal is observed in either cell. Scale bar = 5µm.
